# Predictors of prolonged hospitalization after vaginal birth in Ghana: A comparative study

**DOI:** 10.1371/journal.pgph.0000100

**Published:** 2022-01-05

**Authors:** Samuel Kwaku Essien, Batholomew Chireh, Kidest Getu Melese, John Kwasi Essien

**Affiliations:** 1 School of Rehabilitation Science, University of Saskatchewan, Saskatoon, Saskatchewan, Canada; 2 Saskatchewan Cancer Agency, Saskatoon, Saskatchewan, Canada; 3 Department of Midwifery, College of Health Sciences and Medicine, Wolaita Sodo University, Wolaita Sodo, Ethiopia; 4 Essikado Hospital, Takoradi, Western Region, Ghana; Babcock University, NIGERIA

## Abstract

Early discharge after child delivery although indispensable, but maybe precluded by several factors. The effect of these factors on prolonged length of stay (LOS) after vaginal delivery has been sparsely investigated in Ghana. This limits understanding of potential leading indicators to inform intervention efforts and optimize health care delivery. This study examined factors associated with prolonged LOS after vaginal birth in two time-separated cohorts in Ghana. We analyzed data from Ghana’s demographic and health surveys in 2007 and 2017. Our comparative analysis is based on subsamples in 2007 cohort (*n* = 2,486) and 2017 cohort (*n* = 8,065). A generalized estimating equation (GEE) with logistic regression was used to examine predictors of prolonged LOS after vaginal delivery. The cluster effect was accounted for using the exchangeable working correlation. The odds ratios (OR) and 95% confidence interval were reported. We found that 62.4% (1551) of the participants in 2007 had prolonged LOS after vaginal delivery, whereas the prevalence of LOS in the 2017 cohorts was 44.9% (3617). This constitutes a 17.5% decrease over the past decade investigated. Advanced maternal age (AOR = 1.24, 95% Cl 1.01–1.54), place of delivery (AOR = 1.18, 95% Cl 1.02–1.37), child’s size below average (AOR = 1.14; 95% Cl 1.03–1.25), and problems suffered during/after delivery (AOR = 1.60; 95% Cl 1.43–1.80) were significantly associated with prolonged (≥ 24 hours) length of hospitalization after vaginal delivery in 2017. However, among variables that were available in 2007, only those who sought delivery assistance from non-health professionals (AOR = 1.89, 95% CI: 1.00–3.61) were significantly associated with prolonged LOS in the 2007 cohort. Our study provides suggestive evidence of a reduction in prolonged LOS between the two-time points. Despite the reduction observed, more intervention targeting the identified predictors of LOS is urgently needed to further reduce post-vaginal delivery hospital stay. Also, given that LOS is an important indicator of medical services use, an accurate understanding of its prevalence and associated predictors are useful in assessing the efficiency of hospital management practices and the quality of care of patients in Ghana.

## Introduction

Although there is no consensus regarding the exact length of stay (LOS) after delivery, WHO recommends that, all women who gave birth through vaginal delivery should remain admitted in hospitals or health facilities for a minimum of 24 hours postpartum for observation [1]. This recommendation is to provide ample time for mothers and newborns, especially in low-income countries to be appropriately monitored by skilled birth attendants if a serious postpartum complication arises [1]. The WHO posited that the most crucial and riskiest period for both expectant mothers and their newborns is the first 24 hours after childbirth [2, 3]. Although the WHO recommended extended hospital LOS in low-income countries, this perspective in high-income countries is almost the reverse given that prolonged hospital LOS has consequences for their health care systems. In the past three decades, most developed countries have advocated for an evolution in their health care systems aimed at reducing unnecessary hospital stay after childbirth [4]. Regardless, the burden of prolonged LOS has been reported in both low and high-income countries [3, 5, 6], which include nosocomial infections for both infant and mother, dissatisfaction with health care services, sustainability of health care systems, stress, maternal sleeping disorders, and breastfeeding issues as well affects family ties [3, 5–7].

Early discharge after child delivery although indispensable but may be precluded by several factors, including place of birth and residence type (rural/urban) [8]. Few recent studies however found that extended length of hospital stay is also associated with socioeconomic, demographic, and care factors such as higher socioeconomic status and education, having a private payor source, chronic hypertension, advanced maternal age, obstetric morbidity, mode of delivery, and type of facility [8, 9]. Others also found that regardless of age, women with good insurance policies were more likely to stay for long after birth [10].

Few studies in Ghana have attempted to assess the predictors of LOS [11, 12], although valuable, these studies did not compare prolonged LOS between two time-separated cohorts, amalgamated both delivery types (cesarean section vs vaginal birth) into a single cohort irrespective of their differences, and had small sample sizes not representative of all geographical regions in Ghana, hence precluding generalization of findings. Therefore, the present study is an attempt to fill in these research gaps by exploring differences in predictors of prolonged LOS by comparing two different cohorts over 10 years. This study specifically aimed to 1) estimate differences in the prevalence of prolonged LOS after vaginal birth between two national population cohorts of women of reproductive age; 2) determine whether predictors of prolonged LOS changed over time.

## Methods

### Data sources

**Fig 1:**
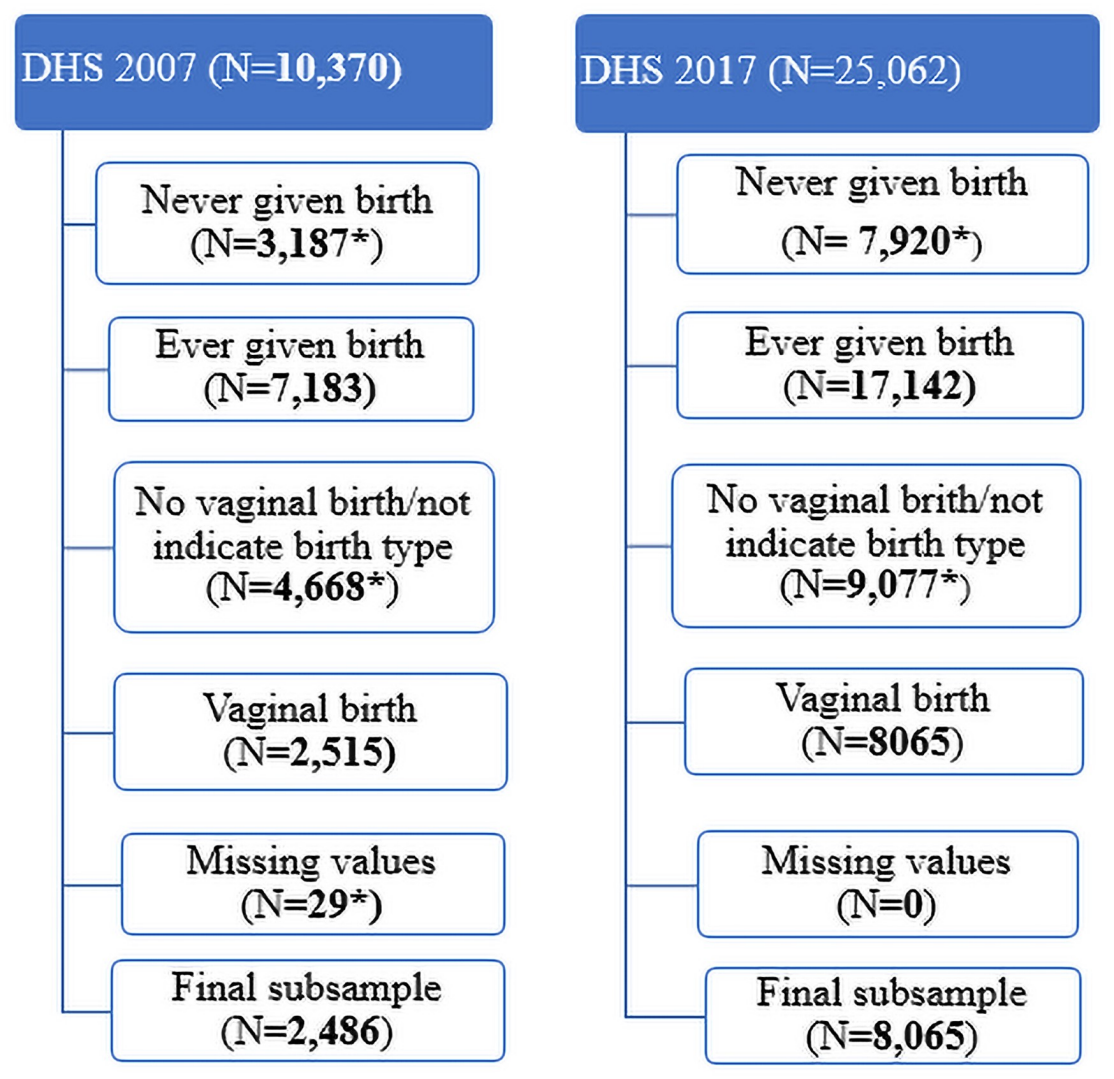
Sample derivation of 2007 and 2017 study samples.

The cross-sectional data for 2007 and 2017 Ghana maternal health surveys were used [13, 14]. These surveys collected nationally representative population-based maternal health indicators including mother and postnatal characteristics and other obstetric care-related emergency events at birth [13, 14]. Both surveys used a two-stage stratified cluster sampling technique to collect data at the cluster, household, and individual levels in 2007 and 2017 [13, 14]. Although data were collected on 10,370 women aged 15–49 years in 2007 from 400 clusters (yielding a response rate of 98%) in 10,858 households [13], 7183 women reported having ever given birth with 2515 through vaginal delivery. For the 2017 survey, data were collected from 25,062 women aged 15–49 years from 900 clusters in 26,324 households [14]. Of the 25062 women, 17,142 reported to had ever given birth with 8065 via vaginal deliveries. We excluded missing data of less than 5% according to Harrell’s recommendation [15]. Thus, the 29 missing data from the 2007 vaginal delivery representing (1.2%) were not included in the study analyses. Fig 1 below provides a detailed description of the criteria used to obtain the subsamples of the DHS 2007 and 2017 among women of reproductive age in Ghana (samples excluded from the analysis are flagged asterisk (*)). The selection of potential predictors for the current study was guided by both Cegolon et al. (2019) [16] and Schorr E. (2012) [17] conceptual/theoretical framework. Variables proposed in both frameworks to be accounted for (if available) in predicting length of stay after delivery include child’s size, maternal age, type of delivery, residence (rural/urban), and health insurance [16, 17].

## Measurements

### Outcome variable

The outcome variable for the present study was the length of stay/length of hospitalization after vaginal delivery and was measured as a binary outcome based on the World Health Organization’s recommended early discharge after vaginal delivery within 24 hours (shorter length hospitalization) and exceeding 24 hours referred to as prolonged length of hospitalization.

### Predictors in both 2007 and 2017 surveys

Several demographic variables including age, place of delivery, place of residence, and education and assistance at delivery were considered. Age was categorized into four groups (15–19 years, 20–24 years, 25–29 years, 30+ years) and education into four levels (no education, primary, Junior High School/Middle (JHS/Middle), Senior High School/higher (SHS/Higher)). Also, place of delivery was categorized into two levels (private facility and government facility), place of residence into two groups (rural/urban), and assistance at delivery into three categories (Health professionals, Non-health professionals, and those who had assistance from both Health professionals and Non-health professional).

### Additional predictors in only 2017 survey

Besides the common variables found in both the 2007 and 2017 surveys, the following factors were also assessed from the 2017 survey. These include problems suffered during/after delivery (no/yes), health insurance (no/yes), and complications detected during antenatal care (no/yes). In addition, a child’s size at birth was categorized into three groups: above average (very large and large size), average, below average (small and very small size) [18].

## Statistical analysis

A generalized estimating equation (GEE) with logistic regression was performed to assess the predictors of prolonged (>24 hours) after vaginal delivery in Ghana [19, 20]. The GEE was employed to model the cluster effect in the data using the exchangeable working correlation structure [19]. An unadjusted analysis was first carried out where predictors with a p-value<0.25 were included as a candidate for the adjusted model [21]. The predictors for the final adjusted model were selected using the backward elimination method where all predictors that met the p-value < 0.05 criterion were retained in the final adjusted model [21]. Possible potential interaction and confounding predictors were assessed against published guidelines in literature [22]. All analyses were performed using SAS 9.4 and the odds ratios and 95% confidence intervals were reported.

## Results

Table 1 presents the characteristics of the study population in the 2007 and 2017 surveys. In 2007, of the 2486 women who had vagina delivery, the majority 1551 (62.4%) had prolonged length of hospitalization while 935 (37.6%) had shorter length hospitalization. However, in 2017, 8065 women reported vagina delivery, of which shorter length hospitalization made up the largest share of the study subjects 4442 (55.1%) while those who had prolonged length of hospitalization constituted 44.9%. Interestingly, the age distribution in the two study years (2007 and 2017) were similar, with more than 75% of women in the age group 25–49 years. In addition, more than two-thirds of women in both 2007 (77.3%) and 2017 (89.9%) delivered in a government health facility.

**Table 1:** Characteristics of the study population in 2007 and 2017.

Variables	DHS 2007 (N = 2486)	DHS 2017 (N = 8065)
	N (%)	N (%)
**Length of Stay**		
Shorter LOS (≤24 hours)	935 (37.6)	4442 (55.1)
Prolonged LOS (≥ 24 hours)	1551 (62.4)	3617 (44.9)
**Age/years**		
15–19	113 (4.6)	396 (4.9)
20–24	483 (19.4)	1553 (19.3)
25–29	616 (24.8)	2000 (24.8)
30+	1274 (51.2)	4116 (51.0)
**Place of delivery**		
Private facility	565 (22.7)	811 (10.1)
Government facility	1921 (77.3)	7254 (89.9)
**Child’s size at birth**		
Above average	N/A	3378 (41.9)
Average	N/A	3162 (39.2)
Below average	N/A	1392 (17.3)
**Problems suffered during/after delivery**		
No	N/A	6717 (83.3)
Yes	N/A	1348 (16.7)
**Health Insurance**		
No	N/A	662 (8.2)
Yes	N/A	7403 (91.8)
**Place of residence**		
Rural	1049 (42.2)	4191 (52.0)
Urban	1437 (57.8)	3874 (48.0)
**Education**		
No education	516 (20.8)	2395 (29.7)
Primary	519 (20.9)	1363 (16.9)
JHS/Middle	1155 (46.4)	2777 (34.4)
SHS/Higher	296 (11.9)	1530 (19.0)
**Complications detected during antenatal care**		
No	N/A	1135 (14.1)
Yes	N/A	6877 (85.3)
**Assistance at delivery**		
Health Professional	2426 (97.6)	7996 (99.1)
Non-Health Professional	41 (1.7)	37 (0.5)
Both	19 (0.7)	32 (0.4)

2017*: Missing Child’s size at birth 133 (1.6%); Complications detected during antenatal care 53 (0.6%).

The largest proportion of women in the 2007 study cohort resided in the urban area (57.8%) whereas, in the 2017 cohort, women who resided in the rural area formed the highest proportion. Approximately, 79% and 70% of the 2007 and 2017 cohorts respectively reported having had formal education. Moreover, almost all (99.1%) women in the 2017 cohort sought delivery assistant from health professionals, with a decline in proportion observed in women who sought delivery assistant from non-health professionals compared to the 2007 cohort.

Also, exploration of the variables available in only the 2017 survey revealed that more than 80% of babies delivered through the vagina had an average and above-average body weight. Approximately 92% of women had health insurance, with 85.3% having their pregnancy complications detected during antenatal care. Further, only a few women (16.7%) reported health problems suffered during/after delivery.

The unadjusted model results in Table 2, which compares the explanatory variables found in both the 2007 and 2017 surveys revealed that only place of delivery was significantly associated with prolonged length of hospitalization (P<0.05) in both years. Whereas, assistance sought at delivery predicted prolonged length of hospitalization in 2007 (p = 0.019), maternal age-predicted prolonged length of hospitalization after vagina delivery (p = 0.029) in 2017. The remainder of the explanatory variables in the unadjusted model was not significantly associated with prolonged length of hospitalization after vagina delivery.

**Table 2:** Comparison of predictors of prolonged length of stay for vaginal delivery, 2007 and 2017.

	DHS 2007			DHS 2017		
Variables	Unadjusted Model		Adjusted Model	Unadjusted Model		Adjusted Model
	OR (95%Cl)	Overall P-value	AOR(95%Cl)	OR (95%Cl)	Overall P-value	AOR(95%Cl)
**Age/years**		0.884			0.029	
15–19	1			1		1
20–24	1.04 (0.82–1.32)			0.92 (0.81–1.05)		0.91 (0.79–1.03)
25–29	0.99 (0.81–1.22)			0.96 (0.86–1.07)		0.94 (0.85–1.05)
30+	1.13 (0.75–1.73)			1.24 (1.01–1.52)		1.24 (1.01–1.54)**
**Place of delivery**		0.049			0.014	
Private facility	1		1	1		1
Government facility	1.23 (1.01–1.50)		1.16 (0.94–1.42)	1.20 (1.04–1.39)		1.18 (1.02–1.37)**
**Place of residence**		0.569			0.925	
Rural	1			1		
Urban	0.93 (0.74–1.18)			0.99 (0.87–1.13)		
**Education**		0.507			0.706	
No education	1			1		
Primary	1.07 (0.80–1.42)			1.03 (0.90–1.18)		
JSS/Middle	1.02 (0.83–1.27)			0.99 (0.87–1.13)		
SSS/Higher	0.88 (0.69–1.12)			0.96 (0.85–1.07)		
**Assistance at delivery**		0.019			0.172	
Health Professional	1		1	1		
Non-Health Professional	2.15 (1.14–4.07)		1.89 (1.00–3.61)	1.59 (0.82–3.08)		

AOR = Adjusted odds Ratio; CI = Confidence Interval.

After controlling for the effect of other explanatory variables in 2007 (Table 2), only assistance at delivery significantly predict the prolonged length of hospitalization after vagina delivery (AOR = 1.89, 95% Cl 1.00–3.61). Regardless, the results showed a higher odds of prolonged length of hospitalization after vagina delivery in women who delivered in a government health facility (AOR = 1.16, 95% Cl 0.94–1.42).

In contrast, the adjusted model in 2017 shows that place of delivery and women aged 30+ years significantly predicted prolonged length of hospitalization after vagina delivery. Thus, the odds of prolonged length of hospitalization after vagina delivery was 1.18 times more likely in women who delivered in government facilities (AOR = 1.18, 95% Cl 1.02–1.37) than those who delivered in a private health facility. Compared to women aged 15–19 years, women aged 30+ years were 1.24 higher odds of prolonged length of hospitalization after vagina delivery (AOR = 1.24, 95% Cl 1.01–1.54). No significant difference was found between women aged 20–24 years and 15–19years (AOR = 0.91, 95% Cl 0.79–1.03), and 25–29 and 15–19 years (AOR = 0.94, 95% Cl 0.85–1.05).

Table 3 summarizes the model results of only explanatory variables and their association with prolonged length of hospitalization after vagina delivery in the 2017 cohort. The adjusted model results show that women with child’s size at birth below average had 1.14 times higher odds of prolonged length of hospitalization after vagina delivery than those with child’s size above average (AOR = 1.14; 95% Cl 1.03–1.25). In contrast, no significant difference was found between women with average child’s size at birth and those with child’s size above average (AOR = 1.02; 95% Cl 0.91–1.15). In addition, women who had health problems during/after delivery were more likely to have prolonged length of hospitalization after vaginal delivery than those who did not (AOR = 1.60; 95% Cl 1.43–1.80). Moreover, women who had health insurance were less likely to have prolonged length of hospitalization compared to those without health insurance (AOR = 0.82; 95% Cl 0.69–0.96).

**Table 3:** Predictors of prolonged length of stay for vaginal delivery, 2017.

	DHS 2017		
Variables	Unadjusted Model		Adjusted Model
	OR (95%Cl)	Overall P-value	AOR (95%Cl)
**Age/years**		0.029	
15–19	1		1
20–24	0.92 (0.81–1.05)		0.91 (0.79–1.03)
25–29	0.96 (0.86–1.07)		0.94 (0.85–1.05)
30+	1.24 (1.01–1.52)		1.24 (1.01–1.54)**
**Place of delivery**		0.014	
Private facility	1		1
Government facility	1.20 (1.04–1.39)		1.18 (1.02–1.37)**
**Child’s size at birth**		0.030	
Above average	1		1
Average	1.02 (0.91–1.15)		1.00 (0.89–1.13)
Below average	1.14 (1.03–1.25)		1.13 (1.02–1.24)**
**Problems suffered during/after delivery**		<0.001	
No	1		1
Yes	1.61 (1.43–1.80)		1.60 (1.43–1.80)**
**Health Insurance**		0.027	
No	1		1
Yes	0.84 (0.71–0.98)		0.82 (0.69–0.96)**
**Place of residence**		0.925	
Rural	1		
Urban	0.99 (0.87–1.13)		
**Education**		0.706	
No education	1		
Primary	1.03 (0.90–1.18)		
JSS/Middle	0.99 (0.87–1.13)		
SSS/Higher	0.96 (0.85–1.07)		
**Complications detected during antenatal care**		0.488	
No	1		
Yes	0.96 (0.84–1.09)		

** Statistical significant.

We found that those with advanced maternal age 30+ years were 1.24 (AOR = 1.24, 95% Cl 1.01–1.54) times more likely to have prolonged length of hospitalization after vaginal delivery than women aged 15–19 years. Further, prolonged length of hospitalization after vagina delivery differed by place of delivery, with women who delivered in a government health facility were more likely (AOR = 1.18, 95% Cl 1.02–1.37) to have an extended stay after vagina delivery than their counterparts who delivered at a private health facility. The remaining variables such as place of residence, education, and complications detected during antenatal care were found not to be statistically significantly associated with prolonged length of hospitalization after vagina delivery in the 2017 cohort.

## Discussion

In the ten years (2007–2017), we found that LOS after vaginal delivery has changed from most of the women having prolonged length of hospitalization in 2007 (62.4%) to the majority having a shorter length of hospitalization in 2017 (55.1%). Advanced maternal age, place of delivery, child’s size below average, and problems suffered during/after delivery were significantly associated with prolonged (≥ 24 hours) length of hospitalization after vaginal delivery in 2017, except for women with health insurance who had lower odds of shorter length of hospitalization.

In contrast, only those who sought delivery assistant from non-healthcare professionals significantly predicted prolonged length of hospitalization after vagina delivery (AOR = 1.89, 95% Cl 1.00–3.61) in the 2007 cohort. Regardless, the results showed a higher odds of prolonged length of hospitalization after vagina delivery in women who delivered in a government health facility. Moreover, the 2017 study cohort witnessed a significant decline (0.5%) in women who sought delivery assistance from non-healthcare professionals than those who sought delivery assistant from non-healthcare professionals in 2007 (1.7%).

The impact of health insurance coverage globally cannot be underestimated in the improvement of health care delivery and discharge outcomes including length of hospitalization [23, 24]. Although the Ghana health insurance started in 2003 [25], the 2007 DHS data did not capture this variable. This precluded the impact of health insurance utilization on length of hospitalization after vaginal delivery to be assessed in 2007. Fortunately, the 2017 data assessed the use of health insurance in women who delivered through the vagina. We found that women with health insurance coverage were less likely to have prolonged length of hospitalization after vagina delivery. This is positive, as access to the national health insurance scheme (NHIS) eliminates some of the financial barriers that prevent pregnant women with low socioeconomic status from accessing healthcare services including antenatal care [26, 27]. Ameyaw et al. noted that over 78% of pregnant women admitted to the significance of the NHIS to healthcare accessibility in Ghana [26]. Regardless, since the NHIS do not absorb all maternal health service-related cost [27], some women are detained after delivery due to their inability to afford payments [28]. This increases the odds of extended hospital stay after delivery especially for women without health insurance coverage or low-income women. Moreover, poverty has caused a prolonged length of hospital stay after delivery for most women in Africa [28, 29]. Cowgill and Ntambue found that Congolese mothers and their infants were detained between 1 to 30 days after delivery due to unpaid medical bills [29]. Likewise, dozen of mothers and their infants in Cameroon were detained for a month after delivery because of unpaid hospital fees [30]. The present study finding that having health insurance reduces a woman’s odds of prolonged hospital stay after delivery is in line with a previously published study [31]. Mendoza et al. found women who lack health insurance were 1.9 times more likely to have prolonged hospital stays than their counterparts [31].

Advanced maternal age has been identified as a significant determinant of most pregnancy discharge outcomes, which prolonged hospitalization is not an exception [11, 32, 33]. The present study’s results on the 2007 cohort found no significant association between all age groups and prolonged hospitalization after vaginal delivery. The insignificant association could in part be attributed to the inadequate sample size used for the 2007 analysis compared to the sample size used the in 2017 cohort. The results from the 2017 cohort rather revealed that women at their advanced maternal age 30+ years were 1.24 times more likely to have prolonged length of hospitalization after vagina delivery than women aged 15–19 years. This finding was consistent with other earlier published studies [11, 33]. Van otterloo et al. found women aged above 30 years were at 1.49–1.77 times higher risk of prolonged length of stay after both vaginal and cesarean births than those aged 25–29 years [33].

Also, the tendency for women to have a prolonged stay after vaginal delivery may vary depending on where the delivery took place (government/public or private health facility) [8]. Although 2007 did not find a statistically significant difference between prolonged hospitalization in women who delivered in a government health facility and private facility. In contrast, the 2017 cohort, with a much larger sample size predicted prolonged hospitalization after vaginal delivery in a government health facility. The contrasting findings on prolonged hospitalization after vaginal delivery and place delivery in 2007 and 2017 cohorts could partially be explained by the differences in the sample sizes. This makes the association found with the 2017 cohort being the true one. The 2017 findings are supported by the quality of healthcare provided by private health facilities than public/government health facilities in Ghana [34, 35]. More so, the shorter stay after vaginal delivery in private health facilities in Ghana could be due to the high health care cost per patient stay [35]. The present study’s findings on the 2017 cohort contradict findings from a study in India [8]. Kumar and Dhillon found prolonged stay after vaginal delivery in women who delivered in private hospitals than in public hospitals [8]. However, the authors attributed the possible explanation of prolonged stay after vaginal delivery in private hospitals in India to the assurance of privacy for the pregnant woman in labour and availability of extra bed facilities to host a family member in private health facilities, which lacks in public hospitals [8].

Another factor that has been reported in the literature to be associated with prolonged hospital stay after delivery is a child’s size at birth [16, 31]. Our 2017 study results revealed that women with child’s size at birth below average had 1.14 times higher odds of prolonged length of hospitalization after vaginal delivery than those with child’s size above average. This finding concurs with findings from earlier studies [16, 31]. Mendoza et al. found child’s birth size below average (especially, under 2,000g) were 4.2 times higher odds of prolonged hospital stay than their counterparts [31].

Health problems and post-childbirth delivery complications may keep a woman in a health facility longer after delivery. The present study found women who had health problems during/after delivery were more likely to have prolonged length of hospitalization after vaginal delivery than those who did not. Our finding is consistent with earlier published studies [31, 33, 36]. Women with chronic hypertension and puerperal infections were 5.9 and 6.9 times respectively, more likely to have prolonged hospital stay after vaginal delivery [33].

Further, non-healthcare professional service may delay pregnant mothers from receiving timely obstetric care for their pregnancy [37], resulting in most mothers being transported/referred to healthcare professionals in later stages of labour [38], which may necessitate an extended stay after childbirth depending on the severity of the conditions. Sumankuuro et al. found mothers perceived quality care of traditional birth attendants was associated with mothers’ delay in timely use of healthcare professionals [37]. Future studies looking into non-healthcare professional-related length of after delivery could further help clarify the association between non-healthcare professional practice and prolonged length of stay after delivery.

## Strengths and limitations

The major strength of this study is the use of nationally representative population-based samples of women of reproductive ages in Ghana to estimate the prevalence and predictors of prolonged LOS at two points in time a decade apart. In addition, this study used relatively sizeable sample sizes, and a robust statistical method (GEE) to estimate prevalence and predictor variables of LOS after vaginal delivery.

Our study has some limitations despite its strengths. Firstly, due to the cross-sectional nature of the DHS study, causality cannot be inferred. Our findings should therefore be interpreted with caution. Second, the absence of important predictor variables in the 2007 cohort did not allow for a fair comparison between the two cohorts. Although there is a high proportion of women in Ghana who deliver at home [39], however, unavailability of data on length of stay in women who had home deliveries precluded further analysis. Lastly, this study is open to recall bias given that our data is based on women’s self-reported information.

## Conclusion

Our study provides suggestive evidence of a reduction in prolonged hospital LOS between the two-time points and shows that predictors of LOS may have changed over time. Advanced maternal age, place of delivery, child’s size below average, and problems suffered during/after delivery were significantly associated with prolonged (≥ 24 hours) length of hospitalization after vaginal delivery in 2017. However, among variables that were available in 2007, only those who sought delivery assistance from non-health professionals were significantly associated with prolonged LOS in the 2007 cohort.

Given that LOS is an important indicator of medical services use, an accurate understanding of its prevalence and associated predictors are useful in assessing the efficiency of hospital management practices and the quality of care of patients in Ghana. A future longitudinal population-based study that assesses the combined effect of variables found to be associated with prolonged LOS after vaginal delivery while accounting for the influence of non-healthcare practice is warranted. In addition, studies targeting the effectiveness of already existing government maternal interventions are encouraged in the future to establish the extent to which they are meeting the various maternal health needs of women of reproductive age in Ghana.

## References

[1] World Health Organization. WHO recommendations on postnatal care of the mother and newborn. Geneva: World Health Organization. 2013. https://www.who.int/health-topics/maternal-health#tab=tab_1. Accessed 10 June 2021. 24624481

[2] Campbell OM, Graham WJ. Strategies for reducing maternal mortality: getting on with what works. Lancet. 2006;368(9543):1284–99. doi: 10.1016/S0140-6736(06)69381-1 17027735

[3] Campbell OMR, Cegolon L, Macleod D, Benova L. Length of stay after childbirth in 92 countries and associated factors in 30 low- and middle-income countries: compilation of reported data and a cross-sectional analysis from nationally representative surveys. PLoS Med. 2016;13(3): e1001972. doi: 10.1371/journal.pmed.1001972 26954561 PMC4783077

[4] Nilsson IMS, Kronborg H, Knight CH, Strandberg-Larsen K. Early discharge following birth–What characterizes mothers and newborns? Sexual & Rep Healthcare. 2017; 11: 60–68. doi: 10.1016/j.srhc.2016.10.007 28159130

[5] Britton JR, Britton HL, Beebe SA. Early discharge of the term newborn: a continued dilemma. Pediatrics. 1994; 94(3):291–5. 8065852

[6] Hellman LM, Kohl SG, Palmer J. Early hospital discharge in obstetrics. Lancet. 1962; 1:227–232. doi: 10.1016/s0140-6736(62)91185-6 13906286

[7] WHO Europe. Modern health care delivery systems, care coordination, and the role of hospitals. 2012. http://www.euro.who.int/__data/assets/pdf_file/0008/158885/BRU-report-Modernhealth-care-delivery-systems.pdf?ua=1. Accessed 11 June 2021.

[8] Kumar P & Dhillon P. Length of stay after childbirth in India: a comparative study of public and private health institutions. BMC Preg Childbirth. 2020; 20:181. doi: 10.1186/s12884-020-2839-9 32293327 PMC7092556

[9] Marshall AL, Durani U, Bartley A, Hagen CE, Ashrani A, Rose C, et al. The impact of postpartum hemorrhage on hospital length of stay and inpatient mortality: a national inpatient sample–based analysis. Am J Obstet Gynecol. 2017; 217(3):344.e1–6. doi: 10.1016/j.ajog.2017.05.004 28502758

[10] Blumenfeld YJ, El-Sayed YY, Lyell DJ, Nelson LM, Butwick AJ. Risk factors for prolonged postpartum length of stay following cesarean delivery. Am J Perinatol. 2015;32(9):825–32. doi: 10.1055/s-0034-1543953 25594218 PMC4504826

[11] Adebanji A, Adeyemi S, & Gyamfi M. Empirical analysis of factors associated with neonatal length of stay in Sunyani, Ghana. J Pub Health Epi. 2015; 7(3): 59–64. doi: 10.5897/JPHE2014.0679

[12] Puurbalanta R, Adebanji AO, & Anang RC. Poisson Log-linear Analysis of Postpartum Length of Stay. Int J Sci Tech. 2014; 2(12): 82–88.

[13] Ghana Statistical Service (GSS), Ghana Health Service (GHS), and Macro International. 2009. Ghana Maternal Health Survey 2007. Calverton, Maryland, USA: GSS, GHS, and Macro International. https://dhsprogram.com/pubs/pdf/FR227/FR227.pdf. Access 11 June 2021.

[14] Ghana Statistical Service (GSS), Ghana Health Service (GHS), and ICF. 2018. Ghana Maternal Health Survey 2017. Accra, Ghana: GSS, GHS, and ICF. https://dhsprogram.com/pubs/pdf/FR340/FR340.pdf. Accessed 11 June 2021.

[15] Harrell Frank E. Missing Data. In Harrell F. E. (1st Edition), Regression modeling strategies: with applications to linear models, logistic regression, and survival analysis (pp.41–51). New York, Springer. 2001.

[16] Cegolon L, Campbell O, Alberico S, Montico M, Mastrangelo G, Monasta L, et al. Length of stay following vaginal deliveries: A population based study in the Friuli Venezia Giulia region (North-Eastern Italy), 2005–2015. PLoS One. 2019 Jan 3;14(1):e0204919. doi: 10.1371/journal.pone.0204919 30605470 PMC6317786

[17] Schorr E. Theoretical framework for determining hospital length of stay (LOS). InBMC Proceedings 2012 Jul (Vol. 6, No. 4, pp. 1–1). BioMed Central.

[18] Boerma J, Weinstein K, Rutstein S, Sommerfelt A. Data on birth weight in developing countries: Can surveys help? World Health Organization. Bulletin of the World Health Organization. 1996; 74(2): 209–216. 8706237 PMC2486906

[19] Hanley JA, Negassa A, Edwardes MD, Forrester JE. Statistical analysis of correlated data using generalized estimating equations: an orientation. Am J Epi. 2003.15; 157(4):364–75. doi: 10.1093/aje/kwf215 12578807

[20] Hilbe JM. Logistic regression models. CRC press; 2009.

[21] Hosmer David W., Lemeshow S, Rodney X. Sturdivant. Applied logistic regression. New York: Wiley; 2000.

[22] Kleinbaum DG, Klein M. Modeling Strategy for Assessing Interaction and Confounding. In: Logistic Regression. Statistics for Biology and Health. Springer, New York, NY. 2010.

[23] Sriram S, Khan MM. Effect of health insurance program for the poor on out-of-pocket inpatient care cost in India: evidence from a nationally representative cross-sectional survey. BMC Health Serv Res. 2020; 20:839. doi: 10.1186/s12913-020-05692-7 32894118 PMC7487854

[24] Dafny L, Gruber J. Public insurance and child hospitalizations: access and efficiency effects. J Pub Eco. 2005;89(1):109–129. 10.1016/j.jpubeco.2003.05.004

[25] Akweongo P, Chatio ST, Owusu R, Salari P, Tedisio F, Aikins M. How does it affect service delivery under the National Health Insurance Scheme in Ghana? Health providers and insurance managers perspective on submission and reimbursement of claims. PloS one. 2021; 16(3):e0247397. doi: 10.1371/journal.pone.0247397 33651816 PMC7924798

[26] Ameyaw EK, Ahinkorah BO, Baatiema L, Seidu AA. Is the National Health Insurance Scheme helping pregnant women in accessing health services? Analysis of the 2014 Ghana demographic and Health survey. BMC Preg Birth. 2021; 21:201. doi: 10.1186/s12884-021-03651-6 33706716 PMC7953785

[27] Dalinjong PA, Wang AY, Homer CS. Has the free maternal health policy eliminated out of pocket payments for maternal health services? Views of women, health providers and insurance managers in northern Ghana. PLoS One. 2018;13(2):e0184830. doi: 10.1371/journal.pone.0184830 29389995 PMC5794072

[28] Kippenberg JA. Hospital detentions in Africa. In J. A. Kippenberg (Ed.), High price to pay: Detention of poor patients in Burundian hospitals (pp. 17). Human Rights Watch; New York, 2006. https://www.hrw.org/report/2006/09/07/high-price-pay/detention-poor-patients-burundian-hospitals#_ftn34. Accessed 24 May 2021.

[29] Cowgill KD, Ntambue AM. Hospital detention of mothers and their infants at a large provincial hospital: a mixed-methods descriptive case study, Lubumbashi, Democratic Republic of the Congo. Rep Health. 2019: 16:111. https://dx.doi.org/10.1186%2Fs12978-019-0777-7. 10.1186/s12978-019-0777-7 PMC6647063 31331396

[30] Maëva Poulet. Cameroon hospital detains new mothers over unpaid fees. https://observers.france24.com/en/20180813-cameroon-hospital-mothers-babies-detained-fees. Accessed 5 May 2021.

[31] Mendoza TLA, Arias GM, Osorio RMÁ. Factores asociados a estancia hospitalaria prolongada en neonatos [Factors associated with prolonged hospital stay in infants]. Rev Chil Pediatr. 2014;85(2):164–173. doi: 10.4067/S0370-41062014000200005 25697204

[32] Ikeda S, Shibanuma A, Silwal R, Jimba M. Factors associated with the length of stay at health facilities after childbirth among mothers and newborns in Dhading, Nepal: a cross-sectional study. BMJ open. 2021;11(5):e042058. 10.1136/bmjopen-2020-042058 33947724 PMC8098919

[33] Van Otterloo L, Connelly C, Gould J, Abreo A, Main E. Mothers at risk: factors affecting maternal postpartum length of stay. J Per Neo Nurs. 2018; 32(4):303–14. doi: 10.1097/JPN.0000000000000342 29939881

[34] Ayiah-Mensah F, Kwabena MA, Sherif M. Comparative Analysis of Patient Satisfaction between Private and Public Hospital. Am Based Res J. 2016; 5: 11–20.

[35] Adongo AA, Azumah FD, Nachinaab JO. A comparative study of quality of health care services of public and private hospitals in Ghana. J Pub Health. 2021:1–7. https://link.springer.com/article/

[36] Watt S, Sword W, Krueger P. Longer postpartum hospitalization options–who stays, who leaves, what changes? BMC Preg Childbirth. 2005;5(1):13. doi: 10.1186/1471-2393-5-13 16225678 PMC1266374

[37] Sumankuuro J, Mahama MY, Crockett J, Wang S, Young J. Narratives on why pregnant women delay seeking maternal health care during delivery and obstetric complications in rural Ghana. BMC pregnancy and childbirth. 2019 Dec;19(1):1–3. 31337348 10.1186/s12884-019-2414-4 PMC6651920

[38] Mills S, Bertrand JT. Use of health professionals for obstetric care in northern Ghana. Studies in family planning. 2005 Mar;36(1):45–56. doi: 10.1111/j.1728-4465.2005.00040.x 15828524

[39] Dzomeku VM, Duodu PA, Okyere J, Aduse-Poku L, Dey NE, Mensah AB, et al. Prevalence, progress, and social inequalities of home deliveries in Ghana from 2006 to 2018: insights from the multiple indicator cluster surveys. BMC pregnancy and childbirth. 2021;21(1):1–2. 34289803 10.1186/s12884-021-03989-x PMC8296527

